# Exploring the integration of fish powder in school meal programs in Malawi through a food environment lens: acceptability, affordability, and convenience

**DOI:** 10.3389/fnut.2025.1605540

**Published:** 2025-06-23

**Authors:** Molly Ahern, Tijoy Lowore, Mihasina Harinaivo Andrianarimanana, Amenye Banda, Jogeir Toppe, Tinna Ng’ong’ola-Manani

**Affiliations:** ^1^Food and Agriculture Organization of the United Nations (FAO), Fisheries and Aquaculture Division, Rome, Italy; ^2^Department of Food Science and Technology, Lilongwe University of Agriculture and Natural Resources (LUANAR), Lilongwe, Malawi; ^3^Food and Agriculture Organization of the United Nations (FAO), Malawi Country Office, Lilongwe, Malawi

**Keywords:** fish powder, school meals, food environment, nutrition, Malawi

## Abstract

**Introduction:**

Despite recognition that fish is a unique source of essential fatty acids, as well as bioavailable protein and micronutrients that are important for child and adolescent development, fish -and animal-sourced foods more broadly- are often not included in school meal programs in low- and middle-income countries in Sub-Saharan Africa. School meal programs have been promoted for decades for improving educational outcomes, such as reduced absenteeism and increased enrolment, but can also improve food security and nutrition of learners and livelihoods for local producers when foods are sourced locally. Even in countries where fish plays an important role in nutrition and food security, such as Malawi, where it provides 14.2% of available animal protein and employment for 217,000 people, it has not been featured in school menus.

**Methods:**

The objective of this study was to explore the integration of fish products into school meal programs that source foods from local producers in Malawi, by assessing the quality, convenience and affordability dimensions of the school food environment. This was done by conducting 1) acceptability trials amongst schoolchildren aged 6-13 years, 2) assessment of time costs for processing fish powders and 3) ease-of-use for school volunteers to integrate fish powder into school meals, and 4) evaluation of the cost of production of fish powders and their affordability for school meal programs.

**Results:**

We found that fish powder incorporated into various school meal recipes were highly accepted, with approximately 90% of learners consuming over 75% of porridges containing pan-roasted fish powder, regardless of whether learners were from a lakeshore or inland district. This was further supported by the highest sensory ratings for attributes such as smell, taste, and appearance of porridges containing pan-roasted *usipa* powder. While pan-roasting the fish before grinding it into fish powder was more accepted by students, pan-roasting added processing time and costs (in relation to other processing methods) due to the need for fuelwood, raising concerns over economic and environmental sustainability.

**Conclusion:**

Adding animal-source food to school menus is one pathway to fight malnutrition and ensure food security. Fish powder has great potential to contribute to this agenda through school meal programs and should be promoted as an ingredient in school meals in Malawi.

## 1 Background

In public health nutrition, much emphasis is placed on the first 1,000 days as this time is vital for physical and cognitive growth. However, research indicates that child development continues for an additional 7,000 days (1, 2). The majority of school meal programs were designed to meet educational goals, such as increased enrolment and reduced absenteeism. However a growing majority of school meal programs also have nutrition and health objectives (3). The State of School Feeding Worldwide report has called for a critical shift in perspective, suggesting that child health and development strategies should expand beyond the “age-siloed” approach centered on the first 1,000 days, encompassing children’s needs throughout their entire life span (the first 8,000 days) (2). The additional 7,000 days represent a period of sustained growth and development, linking two key stages often targeted by public health nutrition: from conception to age 2 years and during the reproductive years (15–49 years). Children and adolescents typically spend a large portion of this time in school, making it an ideal platform to improve nutrition.

Although there is limited empirical evidence about the broader benefits of school feeding beyond education, these programs can also achieve non-educational objectives, such as providing livelihood opportunities for local producers. When school feeding programs supply children with nutritious, diverse, and locally sourced food from small-scale farmers, they are referred to as home-grown school feeding (HGSF) (4). HGSF has substantial potential to boost local economies and improve livelihoods, helping to advance the goals of eliminating hunger (SDG 2) and eradicating poverty (SDG 1) (4). Acknowledging the importance of enhancing child nutrition and supporting local farmers, the New Partnership for Africa’s Development (NEPAD), now known as the African Union Development Agency–NEPAD (AUDA-NEPAD), adopted HGSF as a flagship program under its food and nutrition security initiative (5). Despite these positive efforts, such programs often lack animal-sourced foods such as fish, which are critical for improving adolescent nutrition (6). Including fish and fish-based products in HGSF programs provides essential micronutrients and animal proteins necessary for cognitive growth and overall health, particularly among adolescents (6).

Fish and other aquatic foods are rich in essential nutrients and are an important part of healthy diets (7). In addition, as a source of animal protein, aquatic foods (particularly small pelagic fisheries and mollusk aquaculture) have the lowest carbon footprint when it comes to production (8). Globally, more than 1 billion low-income people obtain most of their average per capita intake of animal protein from fish (9). Thus, fish is an important source of animal protein for countries that are resource-limited and food deficient (10). Often consumed smoked, dried or as a powder, fish is a critical source of dietary protein and micronutrients for many isolated communities in rural areas (11). Fish may also be the sole accessible and/or affordable source of animal protein for poor households in urban or peri-urban areas in Africa. Nutritionally, fish is an important direct source of protein and micronutrients for millions of people in Africa (11). According to Food and Agricultural Organization (12), fish contributes 17.0 and 14.2% of animal-source protein consumed in Africa and in Malawi, respectively (12). In a 24-h dietary recall including 2,861 households with children under 24 months of age across 10 districts of Malawi, only 39% of children age 6–23 months and adolescent girls consumed animal-source food, with fish (particularly small sized species) being the most commonly consumed (13).

In Malawi, the most consumed fish are small fish species like *Matemba* (*Barbus paludinosus*), *Usipa* (*Engraulicrypris sardella*) and *Utaka* (*Corpadichromis virginalis*) because they are abundant, affordable, culturally acceptable, can be sold in small portions and are easy to preserve and store. Of these species, *usipa* was chosen to be tested for acceptability trials in schools as it is highly available across most seasons and throughout all districts of Malawi and is more easily ground into powder than species like utaka, which tends to be oily. Small freshwater fish species are eaten whole with bones and viscera and hence are rich sources of micronutrients such as vitamin A, vitamin D, several B vitamins, and minerals like iron, zinc, calcium, and selenium, and are good sources of high-quality proteins and lipids with long-chain omega-3 fatty acids which contribute to child development (7, 14). Therefore, small local pelagic fish species form an integral component of people’s daily diet (15). In addition, the small fish species are dense in macro and micronutrients, which are particularly important in mitigating the triple burden of malnutrition in low- and medium-income countries in Africa (16).

The UN Nutrition discussion paper on “The Role of Aquatic Foods in Sustainable Healthy Diets” (17) and the State of Fisheries and Aquaculture 2024 (18) highlighted various strategies for promoting sustainably produced aquatic food products that have potential for improving nutrient intake for nutritionally vulnerable populations, as well as creating equitable livelihood opportunities within aquatic food value chains. One such product is fish powder, which can be formulated from various fish species and fish by-products, processed using local processing methods such as sundrying or smoking, and integrated into local recipes for meals and snacks. Fish powders processed from small sundried and smoked fish have been developed and tested for infant and young child feeding in Zambia and Malawi (19, 20) and Bangladesh (21), and tuna frame powders were developed and tested for school feeding programs in Ghana (22, 23), as well as in snacks designed for ready-to-use therapeutic foods (RUTF) in Cambodia (24, 25), among other countries (26). Various local fish species, byproducts, processing methods, and recipes for incorporating these fish powders into meals or snacks were used across these examples, considering local processing and preparation methods. Many of these studies have focused on quality aspects (through nutrient analysis) or testing acceptability of the product, with less focus on affordability, convenience and other aspects of the food environment. A review conducted by Kennedy et al. (27) noted research gaps in relation to assessment of price and affordability of aquatic foods in low- and middle-income countries, as well as promotion and convenience.

The objective of this study was to explore the integration of fish products into school meal programs that source foods from local producers in Malawi, by assessing the quality, convenience and affordability dimensions of the school food environment. Currently, school meals in Malawi lack animal-sourced food, such as fish, which are not only a rich source of bioavailable protein but also offer a range of vitamins, minerals, and long-changed polyunsaturated fatty acids which are beneficial for cognitive and physical development. Tools from the FAO Toolkit for Incorporating Fish into the Home-Grown School Feeding Programme (28) were adapted to the context in Malawi to explore each of these dimensions. First, we explored the quality dimension of the food environment through testing the acceptability and sensory attributes of fish powders incorporated into local porridge recipes among schoolchildren aged 6–13 years in two schools of Dowa and Mangochi districts in Malawi. Secondly, we explored aspects relating to convenience, through assessing the time involved in processing fish powder for fish processors at one fish processing organization, as well as the ease-of-use for school volunteers tasked with preparing school meals with fish powder at the two schools. Lastly, we assessed affordability of fish powders for school meal programs by first assessing the cost of production of fish powders produced by local producers and comparing this to data reported by 25 schools on cost of ingredients for school meals. We then compared the cost of fish powder to published cost of other ingredients commonly sourced for school meals in Malawi and explore the nutritional benefits of school meals enriched by fish powder. Lastly, we discuss the trade-offs across these dimensions and in relation to sustainability.

## 2 Methods

**Figure 1:**
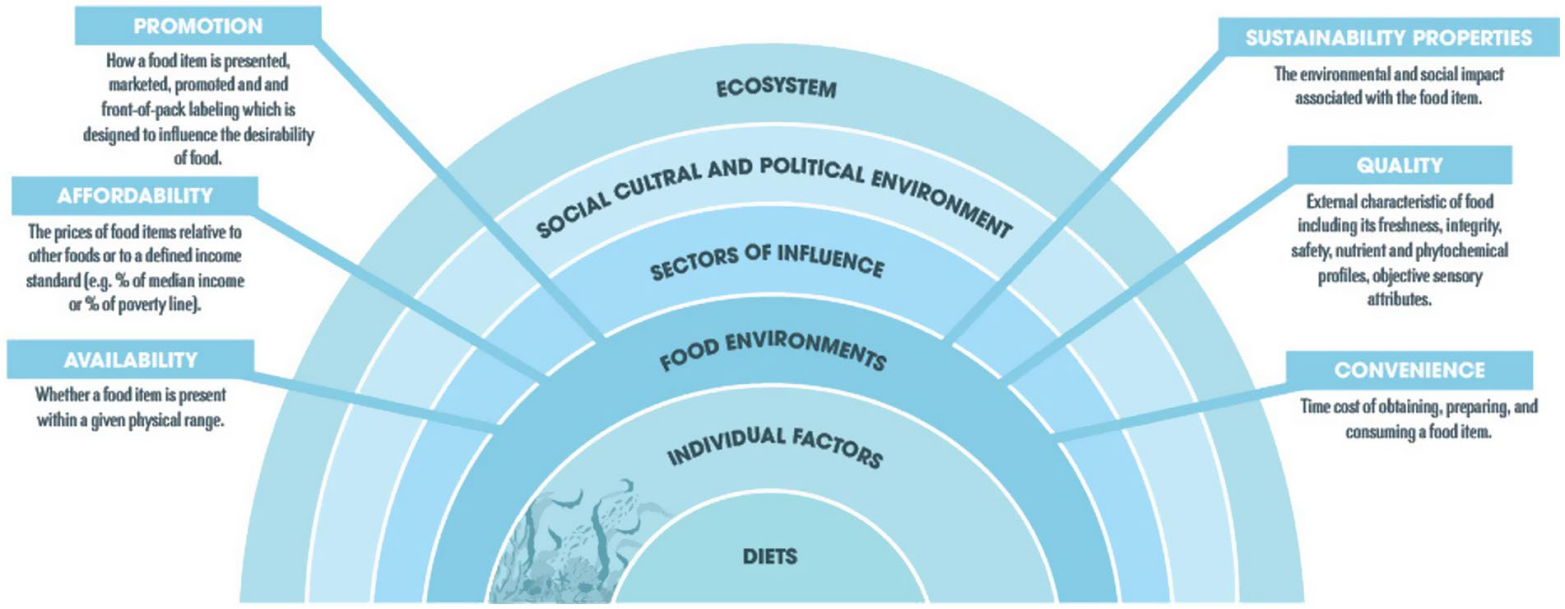
Food environment framework, adapted from Downs et al. (29) and by Kennedy et al. (27), licensed under CC BY 4.0.

In order to integrate fish and fish products from local small-scale producers into school feeding programs, a food environment lens may be beneficial to explore the availability, affordability, promotion, sustainability, quality and convenience of aquatic foods (27). We utilize a conceptual framework of the food environment from Downs et al. (29) (Figure 1) as it is grounded in holistic systems thinking, recognizes sustainability as a characteristic of food environments, is well-suited for multi-disciplinary research and facilitates holistic thinking during program design for interventions that cut across multiple elements of the food environment that influence consumers (27). Affordability is often cited as a key influencing factor for consumers’ food choices (27), however this framework allows for consideration of other factors that may affect consumer choice, or in this case, the choice of ingredients to be included in school meals (which is often driven not only by the consumers’, or students’ choice). We utilized various methods to assess these three dimensions of the school food environment, detailed in Table 1, and described further in the following sections.

**Table 1:** Dimensions of the food environment and methods for assessment used in this study.

Food environment dimension	Method for assessment	Target respondent	Sample size
Quality	Acceptability trials		
	Sensory evaluation	Trained sensory panel	10 trained panelists
	Pre-test for acceptability trials	Schoolchildren (untrained sensory panel)	60 learners (aged 6–13 years) at 1 school in Mangochi and 1 school in Dowa
	Acceptability trials		120 and 116 learners (aged 6–13 years) in 1 school in Mangochi and 1 school in Dowa, respectively
	Plate waste assessment		
Convenience	Time use for production of fish powders	Fish processing organizations	1 fish processing organization (Mangochi District)
	Ease-of-use assessment	School Caterers / Volunteers who prepare school meals	9 volunteers
Affordability	Assessment of cost of production of fish powder	Fish processing organizations	1 fish processing organization (Mangochi District)
	Cost of school meals assessment – to assess cost of fish powder in relation to overall cost of school meals	School Health and Nutrition Coordinator at schools	25 schools across four districts (Mangochi, Dowa, Salima and Karonga)
	Cost of other ingredients used in school meals – to assess cost of fish powder in relation to other ingredients	Published literature	

### 2.1 Assessment of quality dimensions of the school food environment

The quality dimension of food environments includes external characteristics of food, including its freshness, integrity, safety, nutrient and phytochemical profiles, and objective sensory attributes (29). To explore the quality dimension of food environments, we conducted acceptability trials to test fish powders incorporated into porridge recipes served in schools, asking students to try each recipe and evaluate the porridge based on sensory attributes.

Food safety in fish value chains is known to be a challenge in Malawi, and many other lower income countries (30). Microbiological and chemical analyses were conducted, and the results informed the selection of the fish powders that were utilized during the consumer acceptability trials. Although it is an important qualitative dimension of the food environment framework, we do not discuss it at length in this paper. The methods and results of microbiological analysis of fish powders, which informed selection of fish powders for the acceptability trials detailed in this study are presented elsewhere. The below sections describe the experimental design of the acceptability trials.

#### 2.1.1 Acceptability trials

##### 2.1.1.1 Participants

The study targeted primary schools that implement school feeding programs in Mangochi and Dowa districts of Malawi. School-aged children (hereinafter referred to as learners), aged 6–13 years were selected from Msaka Primary School (Mangochi) and Nkhobola Primary School (Dowa) for a pre-test trial. One school in each district was selected for pre-testing as there was a change in the research team in between the two acceptability trials, thus there was need to pre-test with each research team. Learners from Monkey Bay Primary School (Mangochi) and Chilima Primary School (Dowa) were selected as study participants for acceptability trials. Acceptability trials took place in late 2023 in Mangochi and in late 2024 in Dowa. Fish powder was sourced from Kacheta Cooperative, located in Mangochi District, and transported to Monkey Bay Primary School in Mangochi (approximately 3 km) and Chilima Primary School in Dowa (approximately 200-250 km). Fish powder was sourced from the same cooperative for acceptability trials in both districts as the cooperative had capacity to produce the quantity of fish powder needed. The research team transported the fish powder to each of the schools, for the purposes of the acceptability trials.

The sampling was stratified to ensure representation from all sections (junior, middle and senior primary) of the school and participants were randomly selected with the help of the school staff. Learners aged 6–13 years with no known fish, soya beans and groundnut allergens, and that had no infection or congenital disorder, and were capable of swallowing foods were included in the study. Learners with infections such as diarrhea, sporadic cold or fever, sore throat, stomach flu, or headache were excluded from the study. This exclusion criteria was based on known loss of sensory acuity, loss of appetite, and changes in eating habits experienced while sick. Similarly, being on medications also affects sensory acuity. Therefore, learners with such conditions were excluded from the study. Consent from parents or guardians was obtained for all students who participated in the trial. Participants received incentives on days 3 to 5 (up to day 7 in Dowa) of the acceptability trial in order to maintain interest in participation. The protocol for the acceptability trials received ethical clearance (Protocol # 23/06/4132) from the National Health Sciences Research Committee in Malawi.

##### 2.1.1.2 Recipe formulation and meal preparation

The first objective of any public health nutrition intervention is to do no harm. Thus, before conducting the acceptability trials, microbial analysis of the fish powders was conducted to ensure that they were safe to consume. In addition, sensory evaluations with a trained sensory panel were conducted to ensure that recipes were formulated to maximize acceptability and reduce the risk of low consumption, which would jeopardize nutrient intake for children benefiting from school meals.

Prior to the acceptability trial in Mangochi district, only two of the four *usipa* powders tested were deemed safe to consume for the acceptability trial in Mangochi (parboiled and pan-roasted *usipa* powders). Prior to the acceptability trial in Dowa district, a study of critical control points (CCPs) in the *usipa* powder processing cycle was conducted. This identification of CCPs informed a training focused on improving food safety practices in the *usipa* powder processing cycle. Follow-up microbial analysis of *usipa* powders, found that three *usipa* powders were safe for consumption for the acceptability trial in Dowa (parboiled, pan-roasted and sundried).

Sensory evaluations with a trained sensory panel were conducted at Lilongwe University of Agriculture and Natural Resources (LUANAR) in mid-2023. Based on these sensory panels, porridges containing approximately 2.5% fish powder, or 2.5–3.5 g per serving (the amount varied based on the total weight of dry ingredients, see Supplementary Material for detailed recipes) were tested in the acceptability trials to optimize for flavor (smell and taste). During the pre-trial sensory evaluation, an attempt was made to optimize for the provision of nutrient requirements as stipulated in nutritional guidelines and standards (NGS) for school meals in Malawi. In the attempt, nutrient optimization was done using recipe calculation according to Vásquez-Caicedo et al. (55) to meet the recommended dietary intake (RDI). During experimentation, the fish powder inclusion reached up to 5% (based on dry ingredients) in the porridge. However, an initial sensory evaluation with a trained panel revealed that 8 of 10 consumers found the fish flavor to be too strong. Consequently, the optimization prioritized sensory qualities, leading to the selection of a 2.5% fish powder (based on dry ingredients).

In Mangochi, the acceptability trial tested acceptance of *usipa* processed using parboiling and pan-roasting methods. Two recipes normally used in school meals were adapted to include one of two types of fish powder (pan-roasted or parboiled), thus a total of four recipes enriched with fish powder were tested (for example Recipe A with pan-roasted fish powder, Recipe A with parboiled fish powder, Recipe B with pan-roasted fish powder and Recipe B with parboiled fish powder). In addition, a fifth recipe was included as a control, which consisted of the normal school meal without fish powder added (Table 2). Full recipes with ingredients are available in Supplementary Material. A target population of 120 learners belonging to junior, middle and senior classes, and aged 6–13 years from Monkey Bay Full Primary (FP) School in Mangochi participated in the consumer trials as untrained panelists.

**Table 2:** Recipes tested for acceptability in Mangochi and Dowa districts, Malawi.

Mangochi		Dowa	
School recipes	Modified recipes for acceptability trials	School recipes	modified recipes for acceptability trials
Maize-groundnut flour porridge	Parboiled *Usipa* powder-enriched maize-groundnut flour porridge	Maize-groundnut flour porridge	Parboiled *Usipa* powder-enriched maize-groundnut flour porridge
	Pan-roasted *Usipa* powder-enriched maize-groundnut flour porridge		Pan-roasted *Usipa* powder-enriched maize-groundnut flour porridge
			Sundried *Usipa* powder-enriched maize-groundnut flour porridge
Maize-soya flour porridge (control)	Parboiled *Usipa* powder-enriched maize-soya flour porridge	Maize-soya porridge (control)	Parboiled *Usipa* powder-enriched maize-soya flour porridge
	Pan-roasted *Usipa* powder-enriched maize-soya flour porridge	Corn-soya blend (CSB) porridge	Pan-roasted *Usipa* powder-enriched maize-soya flour porridge
			Sundried *Usipa* powder-enriched maize-soya flour porridge

Similarly, in Dowa, a population of 120 learners aged 6–13 years from Chilima Primary School were targeted, however 116 learners took part in the study. Three *usipa* powders were incorporated into the same recipes used for acceptability trials in Mangochi District (maize-groundnut flour porridge and maize-soya flour porridge), resulting in six recipes with fish powder, plus one control recipe (see Table 2). In Dowa district, the school meal program utilizes the central procurement model, in which a fortified corn-soya blend (CSB) is procured centrally and used to make porridge for school meals every day. The same recipes that were used in Mangochi District, based on locally procured ingredients, were used in Dowa, to allow comparison across the two districts. Full recipes with ingredients are available in Supplementary Material.

In both districts, the learners tested one type of porridge per day, in order to avoid fatigue. Sensory panels are prone to fatigue, especially when the number of samples increases, consequently resulting in inconsistent findings (56). The first step during the preparation of the porridges was heating the water until warm. Then, the flours (maize, soya or groundnut and fish) were mixed with warm water. The mixture was stirred frequently to avoid lump formation until a smooth, slightly thick gruel was formed. The gruel was cooked for 45 min on high heat with occasional stirring. Then the porridge was simmered for a further 15 min on low heat. Before serving, 250 g of salt and 500 g of sugar were mixed well with the porridge. The porridge was ready for serving at 6:00 am, the normal mealtime for schools.

##### 2.1.1.3 Presentation of porridge to learners and consumer acceptability assessment

Research assistants at Bunda College, LUANAR were trained on data collection techniques for acceptability trials with young learners prior to the acceptability trial. Following the training, a pre-test was conducted with 60 learners at Msaka FP School in Mangochi district and Nkhobola Primary School in Dowa district. One porridge recipe was tested during pre-testing (maize and soya flour with parboiled *usipa* powder). The pre-test was done to enable the research assistants to familiarize themselves with the questionnaire, experience the challenges arising during data collection from young consumers and envisage the solutions before the actual acceptability trial. Data collected during the pre-test was not included in the analysis.

Porridge was cooked by volunteers at each school (often parents of the children) who are regularly involved in school meal preparation, who were assisted by research assistants in measuring ingredients and serving the porridge. When the porridge was cooked, one scoop of hot porridge equivalent to 437.6 g (± 54.6 g) in Dowa and 294.2 g (± 93.0 g) in Mangochi was poured into transparent containers of the same size and shape, and the containers were closed with fitting lids to keep the porridge warm until it was distributed to the learners. A single scoop of porridge represented the standard serving portion in the schools. Each type of porridge was coded with a 3-digit random number and one type of porridge was presented to the learners for evaluation per day. Transferring the porridge into serving containers for all study participants took 30 min.

**Figure 2:**
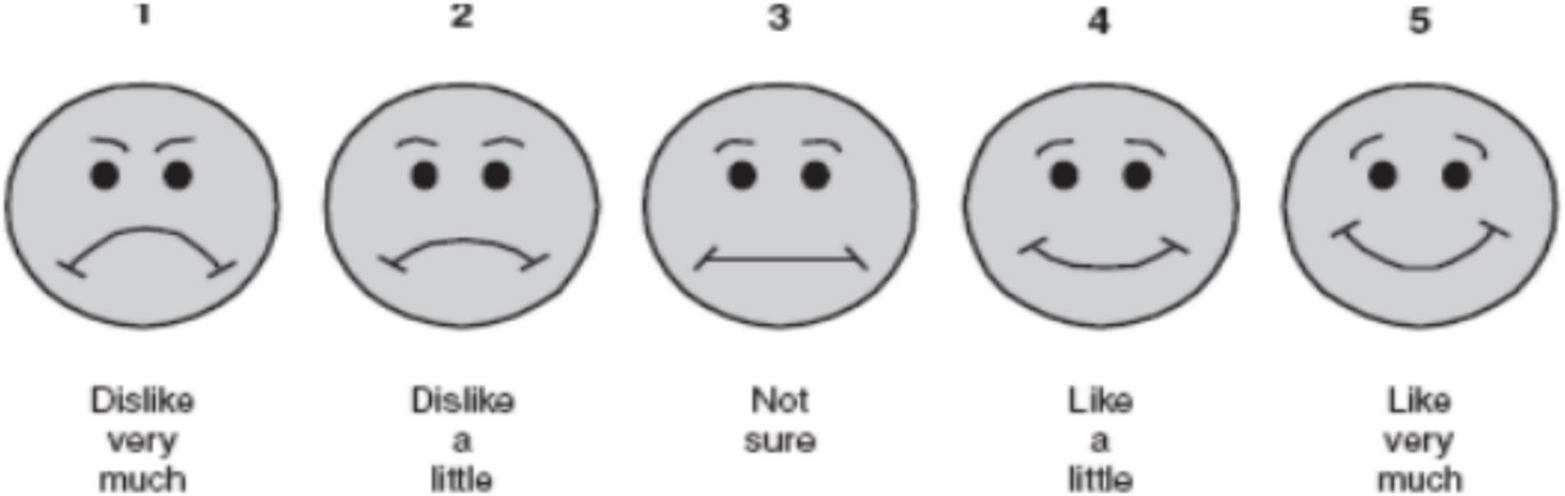
Smiley-meter with 5 point likert scale (28, 54).

For the evaluation of each porridge, each research assistant was assigned a total of 20 learners. The learners tested the porridge in two sessions each day. The first session started from 6:15 am to 7:00 am, with the second session running from 9:00 am to 10:00 am. Sensory attributes such as taste, smell, texture, etc., were evaluated using an enumerator-assisted questionnaire with a 5-point hedonic smiley face scale (Figure 2). The research team clarified the meaning of each category for assessment (smell, taste, appearance, texture, etc.) with learners prior to the evaluation, and research assistants assisted learners during the evaluation with clarifications in order to assist learners with distinguishing between these categories.

##### 2.1.1.4 Statistical analysis of sensory attributes of porridges

The mean and standard deviation of scores for each sensory attribute of each porridge are presented in the results. While it has been suggested by Granato et al. (31) to use median when reporting sensory data, the median score for all sensory attributes for all porridges tested for acceptability in our study was 5. Thus, we have presented the mean and standard deviation here to give more insight into the porridges that were favored by learners.

A pairwise comparison test using Analysis of Variance (ANOVA) was conducted to statistically evaluate the sensory attributes of porridges enriched with fish powder made from dried *usipa*, as perceived by learners in the consumer acceptability trials. The analysis assessed differences between samples, with post-hoc comparisons performed using Tukey’s Honest Significant Difference (HSD) test, as is common with sensory data (31). Mean sensory scores (± standard deviation) for each attribute are reported, and statistically significant differences (*p* < 0.05) are presented in the results.

##### 2.1.1.5 Plate waste assessment

Beyond asking learners to assess sensory attributes of the porridges, plate waste was also measured to determine acceptability. A learner was served with one scoop of porridge in a plastic transparent container. Serving portions were based on the typical practices of each school. In Mangochi, a serving spoon (scoop) was used, while in Dowa, a serving cup was the standard utensil for serving. The porridge prepared from each recipe feeds 120 school learners (± 10). One scoop of porridge was served to each learner each day, with the average scoop weighing 438 g (± 54 g) in Dowa and 294.2 g (± 93.0 g) in Mangochi. The study commenced in Mangochi, where the porridge servings were smaller compared to those in Dowa. At that time of the study starting in Mangochi, the serving sizes used in Dowa were not yet known, so standardization was not applied. The serving sizes were not standardized after learning about the larger serving size in Dowa either, as if we had used the Mangochi serving size in Dowa, we might have inadvertently under-served the learners in comparison to the normal serving sizes they receive at school.

Plate waste evaluation was done according to the procedure described by Hess et al. (32), where learners given the porridge were encouraged to eat and continue eating until full or could not eat any further. Following the procedure set forth by Hess et al. (32), in cases when the taster did not want to eat any more, the taster was given 30 s and then encouraged to consume the food for a second time. If the taster refused to eat, the researcher paused another 30 s. If the taster refuses to eat after the third offer, then the taster is considered to be refusing to eat. Therefore, the leftover (remaining porridge) was weighed to obtain the amount of plate waste in grams (33). The amount of porridge consumed was determined by subtracting the weight of porridge left after eating (plate waste) from the weight of porridge given to the learner before eating. Results are presented as an average weight of porridge served to the learner for each recipe, as well as the average weight of porridge left on the plate after the meal (and the standard deviation). Plate waste was also expressed as a percentage of the amount served. The food is considered acceptable if more than 75% of the served food is consumed (34).

### 2.2 Convenience assessment

The convenience dimension of the conceptual framework of food environments relates to the time cost of obtaining, preparing and consuming a food item (29). The convenience assessments presented here consider the convenience at the production stage of the supply chain for fish powder as well as the convenience of preparing fish powders in school meals. These two stages were considered as any inconvenience for fish processors to produce fish powders or any inconvenience in preparing fish powder-enriched porridges could have an impact on their production and use. The convenience of consuming the fish powder-enriched porridge was determined to be no different than that of the porridges without fish powder, as fish powder was seamlessly integrated into the porridge.

#### 2.2.1 Time involved in processing of fish powder

A tool (28) was developed to assess time costs as well as economic costs in the production of fish powder throughout the value chain, from point of acquisition of raw materials (fresh fish), to point of grinding the fish into powder. This assessment was done for four different fish powder value chains to account for four different processing methods for drying fish before grinding it into powder. These four methods were (1) sundrying, (2) smoking, (3) parboiling and (4) pan-roasting. While the sundrying method is used year-round in Malawi, it works best in the dry season when relative humidity is low (35). Fresh fish is spread on the drying rack (bamboo benches) where solar radiation and open air remove the moisture from fish (36). The method is user-friendly and flexible as it accommodates all types of small fish. For the smoking method, partially sundried fish is smoked using a wire meshed ring onto which the open smoke passes from the smoking kiln, to dry the fish. Smoked fish can be stored throughout the year (36). For the par-boiling method, fresh fish is partially sundried first on the drying rack and dipped in boiling water (100°C) for approximately 2–3 min then sundried again on the drying rack (36). This method is the most often used for processing *usipa*. For the pan-roasting method, partially sundried fish is roasted on dry heat using a metal pan. After pan-roasting, the fish is air-dried on the drying rack.

To collect this data, the research team started by conducting focus group discussions with fish processors. However, as it was difficult to obtain all the information, the research team also collected information using direct observation, where researchers were present during each stage of fish processing at one fish processing cooperative in Monkey Bay, Mangochi District (Kacheta Cooperative). The amount of time for each stage, including purchase at the landing site or wholesaler, transport to the processing center, washing the fish, spreading on drying racks, sundrying, smoking, pan-roasting, or parboiling the fish, and milling the fish into powder was recorded.

#### 2.2.2 Ease-of-use assessment

An ease-of-use assessment was completed at the schools to evaluate the practical implications of incorporating fish powder into school meal recipes. An ease-of-use questionnaire similar to that detailed in Andrianarimanana (28) was used to solicit the opinion of volunteers who are responsible for school meal preparation. These volunteers are community members who are often the parents of children who attend the school. The objective of this assessment was to evaluate if the addition of fish powder as an ingredient in school meals requires any special considerations or additional preparation, as the goal was to not add any burden to the volunteers involved in preparation of school meals. All volunteers were interviewed, including five people from Msaka and Monkey-Bay Primary Schools and four people from Chilima Primary School.

### 2.3 Affordability assessment

The affordability dimension of the conceptual framework for food environments includes the price of food items relative to other foods or relative to a defined income standard (29). To gain this understanding, we first assessed the cost of production of fish powders and compared this to the cost of ingredients for school meals as reported by a random sample of schools that the research team visited in this study. In the discussion, we compare the cost of fish powders to the cost of other ingredients commonly used in school meals in Malawi (costs of groundnut and soya flour based on published literature).

#### 2.3.1 Cost of production of fish powders

The same tool used for evaluating the time cost of the production of fish powders (mentioned in section 2.2.1) was utilized to assess the economic costs of the production of fish powders. This assessment was conducted to assess the costs associated with fish powders produced using four methods of drying fish detailed in section 2.2.1, including (1) sundrying, (2) smoking, (3) parboiling and (4) pan-roasting. To collect this data, the research team conducted focus group discussions as well as direct observation, where researchers were present during each stage of fish processing at one fish processing cooperative in Monkey Bay, Mangochi District (Kacheta Cooperative). Kacheta Cooperative is located on the shore of Lake Malawi, right next to the landing site from which they source fish (approximately 100 m), and approximately 3 km away from Monkey Bay Primary School, where fish powder was tested in acceptability trials.

The costs incurred at each stage were recorded, including purchase at the landing site or wholesaler, transport to the processing center, washing the fish, spreading on drying racks, sundrying, smoking, pan-roasting, or parboiling the fish, and milling the fish into powder. These costs included the cost of raw materials (fresh fish, fuelwood, salt, matches, etc.), maintenance costs for own processing equipment, rental costs for rented processing equipment, and labor. Transportation costs from the fish landing site to the fish processing center were also included, although they were typically minimal as the processing sites were very close to the landing site, thus requiring labor costs for workers to carry the fish. However, transportation costs were not included for distribution from the fish processing center to the schools as this was done by the research team for the purposes of the acceptability trial. In addition, costs relating to storage and marketing were not addressed here as the fish products were directly distributed to schools for the planned acceptability trials.

The quantity (by weight of the fish) at each stage was recorded in order to enable the calculation of the cost per kilogram (kg) for production of fish powder. The research team utilized a scale commonly used in the agricultural supply industry to record weights in kilograms at each stage. These measurements took into consideration the conversion of fresh fish to dried fish (moisture loss) as well as physical losses throughout the value chain (i.e., fish that fell off of the drying rack or kiln trays). It was possible to record most costs in kg, however other costs (such as labor and other inputs such as firewood) were recorded based on the total processing cycle, which is defined here as the time it took to process the initial quantity of fresh fish to fish powder (rather than per hour of labor or per kg of firewood). For maintenance costs of equipment owned by the fish processing organization, these costs were reported per year or biannually.

#### 2.3.2 Exploratory assessment of affordability for school meal programs

The research team visited 25 schools across four districts of Malawi where it is planned to test fish powders in school meals as part of an initial scoping exercise to gain a better understanding of the school food environment across these districts. These districts implement different modalities of school feeding programs, including community-led HGSF (where grants are issued by the government to the school, and the school sources foods locally), HGSF programs implemented by the World Food Programme, and school feeding programs that utilize central procurement models, implemented by Mary’s Meals. Based on enrolment rates and school meal budgets reported by each school (per term), we calculated the amount spent per meal per child and compare the cost of fish powder in relation to the overall amount spent on ingredients for school meals.

## 3 Results

### 3.1 Qualitative assessment — acceptability trials

#### 3.1.1 Sensory evaluation

A total of 223 learners aged 6–13 years assessed the acceptability of dried *usipa* powder-enriched porridges in Mangochi and Dowa districts, however the number of responses for each porridge varied based on attendance each day of the acceptability trial. The study enlisted learners from all sections, junior (Standard 1–2), middle (Standard 3–4), and senior (Standard 5–8) classes. Demographic characteristics of learners who participated in acceptability trials are presented in Table 3.

**Table 3:** Demographic characteristics of learners who participated in acceptability trials.

Characteristics		Frequency (*n*)		Percentage (%)	
		Mangochi (*n* = 120)	Dowa (*n* = 116)	Mangochi	Dowa
Age	4–6 years	14	11	11.67	10.68
	7–9 years	30	39	25.00	37.86
	10–13 years	76	53	63.33	51.45
Sex	Male	57	45	47.50	43.70
	Female	63	58	52.50	56.30
Primary class level	Junior	26	36	21.67	35.00
	Middle	38	39	31.67	37.90
	Senior	56	28	46.67	27.18

The pan-roasted *usipa* powder-enriched porridges consistently received high acceptability scores (i.e., very much liked). In Mangochi, the results on overall acceptability confirmed that 89.17% of learners very much liked (score of 5) the pan-roasted *usipa* powder-enriched maize-soya porridge. The percentage of learners (89.74%) that very much liked the control porridge (i.e., maize-soya porridge) was not significantly different from the percentage of those that very much liked the pan-roasted *usipa* powder-enriched maize-soya porridge. The high acceptability scores of these two porridges confirm their desirability among the target group. However, it is important to note that all the porridges received an mean score of 4.5 and above for their overall acceptability, which means that the majority of learners liked the porridges (either a little – 4, or a lot – 5). In addition to expressing their overall liking of the porridge, the learners also evaluated key sensory attributes of each porridge, including smell, appearance, taste, smoothness, and texture (Table 4). This sensory evaluation is crucial for understanding consumer preferences among this age group to help guide product improvement.

**Table 4:** Results from acceptability trials in Mangochi and Dowa.

Porridge	Trial site	Aroma	Appearance	Taste	Smoothness	Texture	Overall acceptability
1. Maize-soya flour porridge (control)	Mangochi (*n* = 117)	4.70 ± 0.67^ab^	4.65 ± 0.66^ab^	4.66 ± 0.73^ab^	4.65 ± 0.80^ab^	4.62 ± 0.79^ab^	4.87 ± 0.41
	Dowa (*n* = 101)	4.80 ± 0.60^b^	4.58 ± 0.67^a^	4.68 ± 0.72^ab^	4.65 ± 0.64^ab^	4.63 ± 0.69^ab^	4.60 ± 0.76
2. Parboiled *Usipa* powder-enriched maize-soya flour porridge	Mangochi (*n* = 95)	4.58 ± 0.89^a^	4.45 ± 0.93^a^	4.34 ± 0.94^a^	4.48 ± 0.98^a^	4.40 ± 1.04^a^	4.65 ± 0.74
	Dowa (*n* = 103)	4.49 ± 0.99^a^	4.34 ± 1.00^a^	4.41 ± 0.85^a^	4.38 ± 1.10^a^	4.46 ± 1.05^a^	4.59 ± 0.81
3. Pan-roasted *Usipa* powder-enriched maize-soya flour porridge	Mangochi (*n* = 120)	4.71 ± 0.72^ab^	4.73 ± 0.60^b^	4.65 ± 0.72^ab^	4.68 ± 0.78^ab^	4.78 ± 0.55^b^	4.71 ± 0.41
	Dowa (*n* = 101)	4.70 ± 0.76^ab^	4.54 ± 0.74^a^	4.69 ± 0.61^ab^	4.59 ± 0.86^a^	4.66 ± 0.75^ab^	4.75 ± 0.64
4. Sundried *Usipa* powder-enriched maize-soya flour porridge	Mangochi (*n* = 0)	*	*	*	*	*	*
	Dowa (*n* = 102)	4.67 ± 0.69^ab^	4.50 ± 0.76^a^	4.59 ± 0.64^a^	4.44 ± 0.86^a^	4.41 ± 0.87^a^	4.79 ± 0.51
5. Parboiled *Usipa* Powder-enriched maize-groundnut flour porridge	Mangochi (*n* = 117)	4.42 ± 0.96^a^	4.62 ± 0.72^a^	4.56 ± 0.83^a^	4.49 ± 0.90^a^	4.70 ± 0.63^ab^	4.78 ± 0.48
	Dowa (*n* = 97)	4.56 ± 0.98^a^	4.59 ± 0.77^a^	4.62 ± 0.77^ab^	4.54 ± 1.01^a^	4.57 ± 0.90^a^	4.71 ± 0.66
6. Pan-roasted *Usipa* powder-enriched maize-groundnut flour porridge	Mangochi (*n* = 114)	4.79 ± 0.56^b^	4.68 ± 0.71^ab^	4.75 ± 0.60^b^	4.75 ± 0.67^b^	4.81 ± 0.53^b^	4.83 ± 0.46
	Dowa (*n* = 97)	4.57 ± 0.87^a^	4.64 ± 0.70^ab^	4.53 ± 0.79^a^	4.52 ± 0.94^a^	4.75 ± 0.66^b^	4.56 ± 0.83
7. Sundried *Usipa* powder-enriched maize-groundnut flour porridge	Mangochi (*n* = 0)	*	*	*	*	*	*
	Dowa (*n* = 98)	4.59 ± 0.76^ab^	4.50 ± 0.78^a^	4.51 ± 0.86^a^	4.35 ± 0.94^a^	4.43 ± 1.11^a^	4.62 ± 0.67

Reported values represent the Mean and SD, the means in the column with different superscripts were statistically different (*p* < 0.05) based on pairwise comparisons. Scoring was based on a five (5) point hedonic scale where 1–5: 1 = Dislike very much, 2 = dislike a little, 3 = not sure, 4 = like a little and 5 = like very much.

^a, b^The means with different superscripts were statistically different (*p* < 0.05) based on pairwise comparisons.

^*^Sundried *usipa* powder-enriched porridges were not tested in Mangochi District due to food safety issues.

As Table 4 indicates, there were significant differences (*p* < 0.05) in the degrees of liking for all of the sensory attributes. In Mangochi, the *smell* of the pan-roasted *usipa* powder-enriched maize-groundnut porridge received the highest mean score (4.79), followed by the pan-roasted *usipa* powder-enriched maize-soya porridge (4.71), maize-soya control porridge (4.70), and the parboiled *usipa* powder-enriched maize-groundnut porridge (4.58). In Dowa, the *smell* of the maize-soya control porridge received the highest mean score (4.80), followed by the pan-roasted *usipa* powder-enriched maize-soya porridge (4.70), sundried *usipa* powder-enriched maize-soya porridge (4.67), sundried *usipa* powder-enriched maize-groundnut porridge (4.59), pan-roasted *usipa* powder-enriched maize-groundnut porridge (4.57), parboiled *usipa* powder-enriched maize-groundnut porridge (4.56), and the parboiled *usipa* powder-enriched maize-soya porridge (4.49).

In Mangochi, the *appearance* of the pan-roasted *usipa* powder-enriched maize-soya porridge received the highest mean score (4.73), followed by the pan-roasted *usipa* powder-enriched maize-groundnut porridge (4.68), and the maize-soya control porridge (4.65). In Dowa, the *appearance* of the pan-roasted *usipa* powder-enriched maize-groundnut porridge received the highest mean score (4.64), followed by the parboiled *usipa* powder-enriched maize-groundnut porridge (4.59) and the maize-soya control porridge (4.58).

In Mangochi, the *taste* of the pan-roasted *usipa* powder-enriched maize-groundnut porridge received the highest mean score (4.75), followed by the maize-soya control porridge (4.66), and the pan-roasted *usipa* powder-enriched maize-groundnut porridge (4.65). In Dowa, the *taste* of the pan-roasted *usipa* powder-enriched maize-soya porridge received the highest mean score (4.69), followed by the maize-soya control porridge (4.68) and the parboiled *usipa* powder-enriched maize-groundnut porridge (4.62).

In Mangochi, the *smoothness* of the pan-roasted *usipa* powder-enriched maize-groundnut porridge received the highest mean score (4.75), followed by the pan-roasted *usipa* powder-enriched maize-soya porridge (4.68), and the maize-soya control porridge (4.65). In Dowa, the *smoothness* of the maize-soya control porridge received the highest mean score (4.65), followed by the pan-roasted *usipa* powder-enriched maize-soya porridge (4.59) and the parboiled *usipa* powder-enriched maize-groundnut porridge (4.54).

In Mangochi, the *texture* of the pan-roasted *usipa* powder-enriched maize-groundnut porridge received the highest mean score (4.81), followed by the pan-roasted *usipa* powder-enriched maize-soya porridge (4.78), and the parboiled *usipa* powder-enriched maize-groundnut porridge (4.70). In Dowa, the *texture* of the pan-roasted *usipa* powder-enriched maize-groundnut porridge received the highest mean score (4.75), followed by the pan-roasted *usipa* powder-enriched maize-soya porridge (4.66), and the maize-soya control porridge (4.63).

Generally, we observed a trend where porridges containing pan-roasted *usipa* received the highest ratings, regardless of whether participants were from a lakeshore or inland district. However, consumers in the lakeshore district of Mangochi rated attributes highest when the porridge included groundnut in addition to pan-roasted *usipa* powder, while participants in the inland district of Dowa gave the highest ratings to porridges containing soya alongside pan-roasted *usipa*.

#### 3.1.2 Plate waste assessment

Plate waste of the porridge per learner was calculated to ascertain the acceptance of the porridges. The average weight of porridge served to each learner (in grams) is detailed in (32). The recipe with the least plate waste in both Dowa and Mangochi was the control porridge (7.8 ± 24.5 g and 13.2 ± 43.2 g, respectively), followed by the pan-roasted *usipa* powder-enriched maize-soya porridge (19.5 ± 64.1 g and 14.6 ± 49.4 g, respectively) (Table 5).

**Table 5:** The average weight of porridge served to each learner, and average weight of porridge left on the plate after consumption (in grams) for each recipe.

Recipe	Mangochi		Dowa	
	Average of weight of food served (g)	Average of weight of food after consumption (g)	Average of weight of food served (g)	Average of weight of food after consumption (g)
1. Maize-soya flour porridge (control)	311.8 ± 58.5	13.2 ± 43.2	432.9 ± 50.0	7.8 ± 24.5
2. Parboiled *Usipa* powder-enriched maize-soya flour porridge	301.9 ± 15.6	17.7 ± 46.5	439.3 ± 68.7	23.9 ± 78.6
3. Pan-roasted *Usipa* powder-enriched maize-soya flour porridge	301.6 ± 75.4	14.6 ± 49.4	444.9 ± 52.3	19.5 ± 64.1
4. Sundried *Usipa* powder-enriched maize-soya flour porridge			426.9 ± 52.8	29.4 ± 74.7
5. Parboiled *Usipa* powder-enriched maize-groundnut flour porridge	267.2 ± 170.9	39.4 ± 89.3	439.5 ± 27.2	19.5 ± 59.6
6. Pan-roasted *Usipa* powder-enriched maize-groundnut flour porridge	288.7 ± 61.9	15.8 ± 43.5	437.6 ± 68.5	24. 9 ± 75.7
7. Sundried *Usipa* powder-enriched maize-groundnut flour porridge			442.6 ± 52.2	15.2 ± 52.1
All Recipes	294.2 ± 93.0	20.1 ± 57.8	437.6 ± 54.6	20.0 ± 63.8

**Figure 3:**
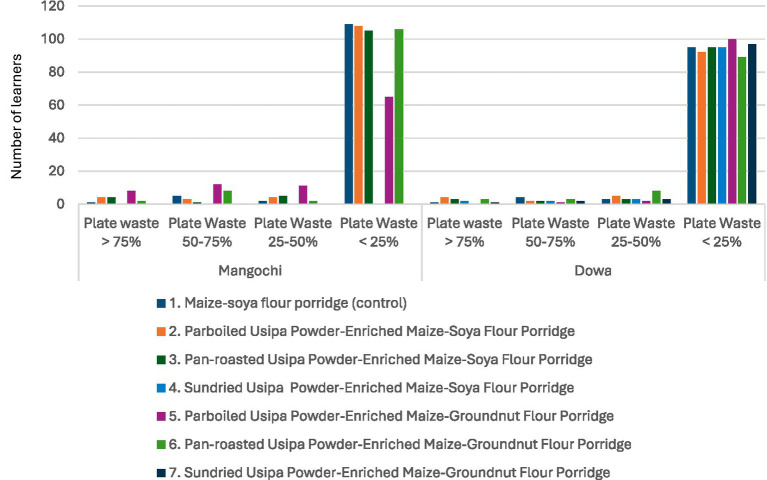
Number of Learners (on *y*-axis) who returned their plate with >75% plate waste, 50–75% plate waste, 25–50% plate waste, and less than 25% plate waste.

According to Hess et al. (32) food is considered accepted if 75% of it is consumed. Across most recipes in both districts, more than 80 percent of learners consumed the majority of the porridge served to them (>75% of the porridge). One porridge was an exception, as only 66% of learners consumed >75% of the parboiled *usipa* powder-enriched maize-groundnut porridge in Mangochi. The other 34% of the learners had >75% plate waste (*n* = 8), 50–75% plate waste (*n* = 20), or 25–50% plate waste (*n* = 11) (Figure 3).

### 3.2 Convenience assessments

#### 3.2.1 Time involved in processing of fish powder

**Figure 4:**
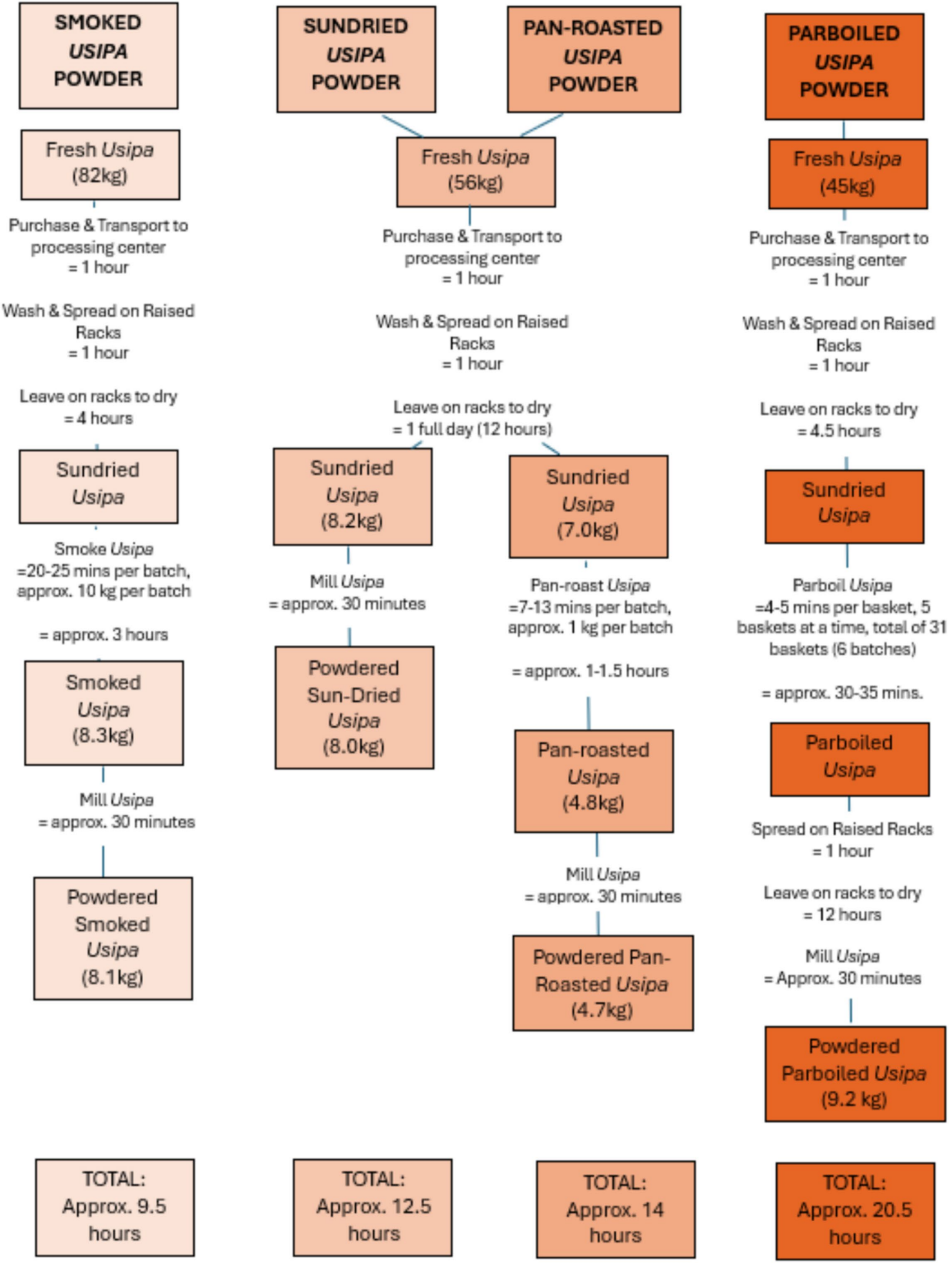
Processing cycle for four different processing methods for fish (usipa) powder and time involved in each step.

The research team recorded each step in the value chain for the processing of four different types of *usipa* powder (smoked, sundried, pan-roasted, and parboiled) through direct observation. Across all four processing methods, the initial steps (purchase at landing site, transport to processing center, washing fish and spreading on raised drying racks) remained the same. However, the amount of time that the fish was left to sundry differed for each method (see Figure 4). Smoked fish powder had the shortest total processing cycle time (9.5 h), while parboiling had the longest (20 h). Sundried *usipa* powder had the least number of steps in the processing cycle, as fish was simply left on raised drying racks without additional processing.

#### 3.2.2 Ease-of-use assessment

The unanimous feedback from volunteers who prepared porridges indicated that the ready-to-use fish powders did not introduce problems during use because the fish powders did not require additional processing prior to preparation of the school meals (porridge). Despite the volunteers’ lack of experience in using fish powders, volunteers found the addition of fish powder to the porridge to be simple. The volunteers affirmed that adding fish powder did not extend the porridge preparation or distribution time. None of the volunteers had prior experience incorporating fish powder into porridges for HGSF. Throughout the consumer acceptability trials’ week, these volunteers participated in mixing fish powders with maize and soya or groundnut while preparing porridge. Consequently, their feedback on ease of use was based on this brief hands-on experience. The volunteers found the fish powders easy to use, attributing this ease to the fact that the powders were provided ready-to-use, eliminating the need for additional processing of the fish. However, due to their lack of prior experience with storing fish powders, the volunteers refrained from providing confident assessments of whether the powders were easy to store or not. The volunteers expressed strong agreement that incorporating fish powder into the recipes did not prolong the time needed to prepare the porridge or distribute the meals. However, they anticipated that the addition of fish powder might increase the overall cost of the porridge, considering the introduction of an extra ingredient.

### 3.3 Affordability assessment

#### 3.3.1 Cost of production of fish powders

**Figure 5:**
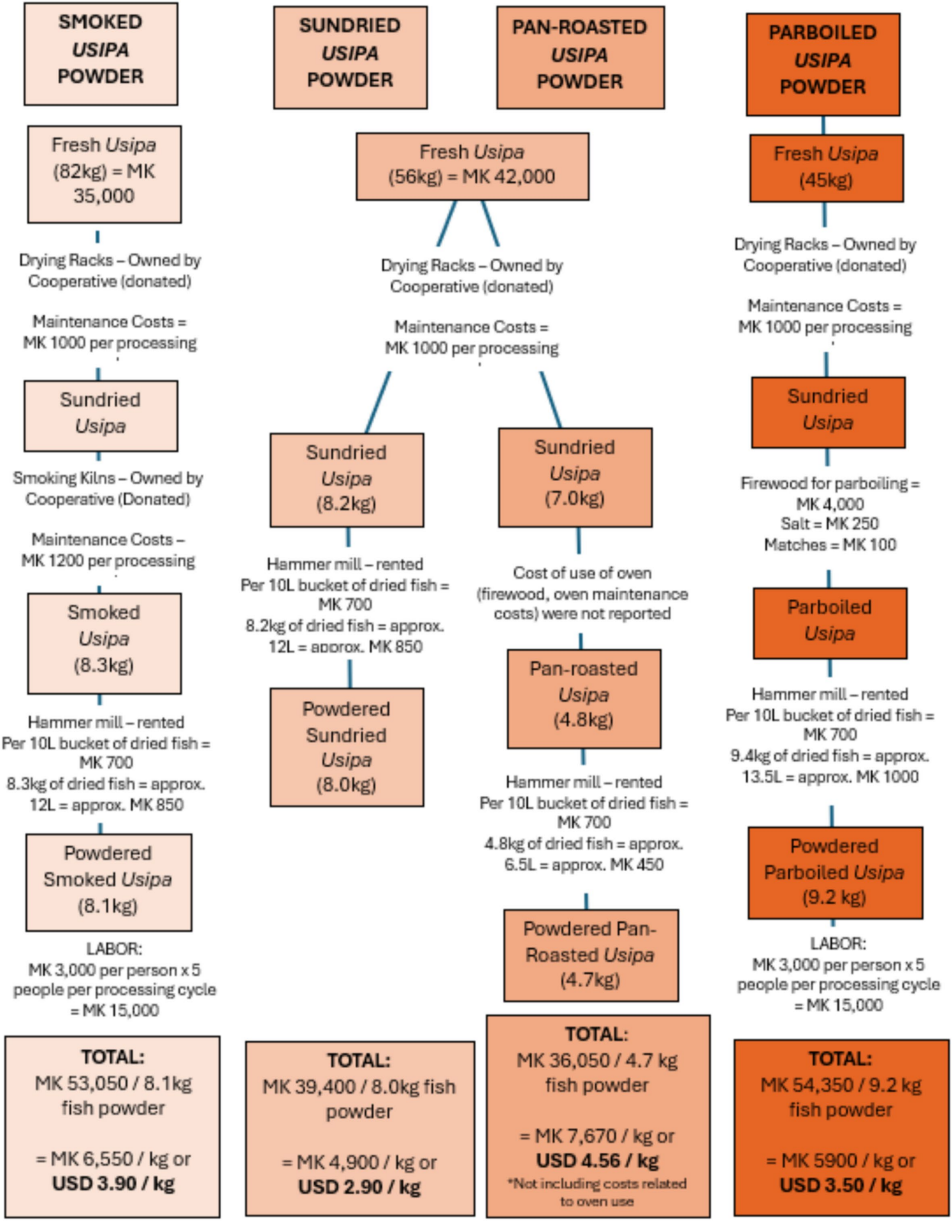
Processing cycle for four different processing methods for fish (usipa) powder and costs involved in each step.

The assessment of production costs of fish powders revealed that expenses were influenced by various factors beyond the cost of fresh fish. For instance, processing methods involving heat treatments such as smoking, parboiling, and pan-roasting were primarily impacted by the cost of fuel, particularly firewood. Salt was an additional input used in the partial boiling step for parboiled usipa powder, however only 100 g of salt was used in parboiling 45 kg of fresh usipa. Among the different methods (Figure 5), open sundrying was the most economical, with a production cost of Malawi Kwacha (MK) 4,900 per kilogram (USD 2.90/Kg). This was followed by parboiled *usipa* at MK 5,900 per kilogram (USD 3.50/Kg). The cost of producing smoked *usipa* powder was MK 6,550 per kilogram (USD 3.90/Kg), while pan-roasted *usipa* had the highest production cost at MK 7,670 per kilogram (USD 4.56/Kg). The cost of pan-roasted fish powder was greater than sundried fish powder in this case as the weight of the end product (pan-roasted *usipa* powder) was much less, thus the costs of production per kilogram of fish powder was higher. This may have been caused by pan-roasting resulting in greater moisture loss (and thus, likely improved shelf life) in relation to the sundried fish powder, or there may have been greater physical loss of fish, which was not recorded (as storage overnight in the school facilities was not monitored). Across all four *usipa* fish powders, there was very little physical loss of fish or fish powder recorded at each stage of processing, thus pointing to moisture loss as the main reason for reduction in weight of the final *usipa* powders. The weight of the final product (*usipa* powder) ranged between 10 and 20% of the weight of the fresh fish used to process the powders.

#### 3.3.2 Analysis of affordability for school meal programs

As part of an initial scoping study, the research team visited 25 schools across 4 districts in Malawi where the integration of fish powder into school meals is being explored. Enrolment rates, amount spent per week (in Malawi Kwacha, MK) and funding source were requested from each school, with 20 of the 25 schools having this information readily available. This data is available in Supplementary Material, and a summary is available below in Table 6.

**Table 6:** Average amount spent per meal, based on 5 meals per week (USD) by funding source for school meal programs across randomly selected schools surveyed in 4 regions of Malawi.

Funding source	Average amount spent per student per week (USD)	Number of Schools *n* total = 20
WFP	$0.40	10
Mary’s meals	$0.35	6
Community-based	$0.20	4

For example, if 2.5 g of fish powder (at lowest unit price calculated here at USD 2.90/kg) were added to school meals three times per week, this would cost approximately 2 cents of one USD (USD 0.02) per student per week. The average cost of school meals per student per week was USD 0.20–0.40 (as collected from the random sample of schools we surveyed), which is consistent with other findings (37). Considering this average cost per child, the addition of fish powder to school meals would represent about 5–10% of the weekly spending on food for school meals. However, as school meal programs in low- and middle-income countries often operate on tight budgets, there may not be flexibility to add ingredients, even if they represent a small percentage of overall spending on meal ingredients. In the discussion, we explore the estimated costs and potential benefits of substituting fish powder with other ingredients commonly used in Malawi’s school meal program.

## 4 Discussion

### 4.1 Quality

The porridges enriched with pan-roasted *usipa* powder were found to be most acceptable to the learners in this study. This finding is consistent with Msiska et al. (38) who reported that products like fish biscuits made with roasted fish powder were highly preferred by the majority of consumers. As explained by Jeong et al. (39), the roasting process enhances the sensory qualities of products, including smell, appearance, and texture, thereby increasing their appeal and stimulating appetite. Jeong et al. (39) further expounded on the chemistry behind roasting, explaining its impact on the chemical composition, physical properties, and sensory properties (e.g., colour, smell, texture) that contribute to an enhanced food experience. Zhang et al. (40) highlighted that the roasting process not only amplifies desirable sensory characteristics but also diminishes the fishy smell or undesirable off-flavors, which may arise during sundrying fish. Research by (39) also underscored the importance the key sensory taste of umami - which is often descirbed as a savory flavor — in influencing the palatability and acceptability of a food. As they are rich in amino acids (such as glutamic acid) and ribonucleic acids [such as guanosine monophosphate (GMP) and inosine monophosphate (IMP)], fish are known to enhance umami even when added to foods in small amounts. Research by Jeong et al. (39) and Oluwaniyi et al., (41) demonstrated that roasting significantly increased the concentration of glutamic acid by 8.9%, GMP by 0.5%, and IMP by 0.5%, respectively. In the present study, the evident preference among learners for the porridges enriched with pan-roasted *usipa* powder can be attributed to these enhancements in sensory qualities and umami characteristics.

The combination of improved sensory properties and the reduction of undesirable flavors through the roasting process contributed to the heightened acceptability of the porridges enriched with the pan-roasted *usipa* powder as compared to those enriched with fish powders prepared using other methods. For example, the porridges enriched with parboiled *usipa* powder received lower acceptability scores among the learners (although they were still in the acceptable range, as they scored at 3 or above). These results align with the findings of Msiska et al. (38), who noted a similar lack of preference among consumers for products incorporating unroasted parboiled *usipa* powder. This observation is consistent with the research of Pasaribu et al. (42) and Oluwaniyi et al. (43), who reported that boiling fresh fish results in the destruction of certain amino acids, with a notable reduction in glutamic acid concentration. The impact of the boiling process on amino acids, particularly the reduction in glutamic acid concentration, was further explored by Ismail and Khairul Ikram (44), revealing a decrease in umami concentration. Taken as a whole, these results suggest that the reduction in glutamic acid negatively impacted consumers’ abilities to detect umami, thereby diminishing the overall taste and acceptance of fish powder-enriched products that did not use pan-roasted fish powder. In essence, the relatively lower acceptability ratings of porridges containing parboiled *usipa* powder can be attributed to the adverse effects of the boiling process on the amino acid composition, particularly the reduction in glutamic acid, ultimately influencing the umami concentration and diminishing the overall sensory appeal and consumer acceptance of the product.

### 4.2 Convenience

In terms of convenience, the sundried *usipa* powder had the least number of steps in the processing cycle, as fish was simply left on raised drying racks without additional processing. Smoked *usipa* powder had the shortest processing cycle (approximately 9 h). However, sundried *usipa* powder and smoked *usipa* powder were not tested in acceptability trials in Mangochi, as issues relating to food safety were found by the laboratory. Further research was conducted by the research team to determine critical control points for improving food safety in the value chain for these products, particularly considering that sundrying fish is a key step in processing all four fish powder products tested. Training was conducted with fish processors to improve the food safety of sundried fish products. Prior to the acceptability trial in Dowa, fish powders made from sundried *usipa* were re-analyzed and found to be safe for consumption.

It was noted by school volunteers that fish powder was easy to add to the morning porridge, as it did not require any additional labor or time and could be easily be incorporated into the porridge. Fish powder is designed to be cooked into porridge or other foods for school meals and thus undergoes a step of heat treatment during preparation (a second step of heat treatment in the case of parboiled, pan-roasted, and smoked fish powders). In theory, if the fish powders processed using parboiling, pan-roasting and smoking methods are done in a hygienic way, the second step of heat treatment at the stage of porridge preparation may not be necessary. However, it was advised to cook the fish powder into the porridge to ensure food safety and even distribution throughout the porridge. The packaging of fish powder should clearly state that the product should be cooked, however further research may consider the optimal amount of time for cooking the fish powder in the porridge mixture to ensure food safety while also optimizing nutrient retention.

### 4.3 Affordability

For the sake of simplicity, the recipes that were tested during the acceptability trials considered fish powder as an additional ingredient rather than an alternative ingredient. This means that the recipes regularly used by the schools were not altered other than *adding* fish powder, rather than *substituting* (in part) the fish powder for another ingredient in the meal. In the case that fish powder would be simply added to existing school recipes as done in the acceptability trials presented here, the cost of the fish powder would be in addition to the regular spending on school meals. However, if considered as a substitute for (part of) other ingredients such as soya or groundnut flours, it becomes more economically feasible for school meal programs to include. For example, if 2.5 g of fish powder (at lowest unit price calculated here of USD 2.90/kg) were substituted for 2.5 g of groundnut flour (at mean unit price MK 2500/kg. or USD 1.42/kg) per student (45) it would result in an increase in cost per student per meal of less than one cent of a USD (USD 0.0037). If fish powder were substituted for a part of groundnut flour 3 times per week, it would cost approximately 1 cent of a USD (USD 0.01) per student per week. Considering the average spending on food for school meals per student per week of USD 0.20–0.40 (as collected from the random sample of schools we surveyed), this would represent about 2.5–5% of the weekly spending on food for school meals.

### 4.4 Trade-offs across these dimensions

#### 4.4.1 Economic sustainability, costs and nutritional benefits

Given the higher cost to produce pan-roasted *usipa* powder, which was noted in our study, it is less likely to be economically sustainable for school meal programs, as they often have strict budget limitations. However, the increased cost of pan-roasted fish powder in relation to the other fish powders related to the reduced weight of the end product, thus the cost per kilogram was greater. This was likely due to extra step of pan-roasting resulting in greater moisture loss, which has likely has additional benefits such as greater nutrient density and improved shelf life. Given that it is likely that the reduced moisture content results in a greater nutrient density of the pan-roasted fish powder (by weight), the quantity of pan-roasted fish powder used to enrich school meals could be reduced while still realizing the same nutritional benefits as the other fish powders presented in this study. However, chemical analysis of fish powders produced using each of these processing methods to provide the full nutrient profile would be beneficial in order to provide the necessary information for optimizing the use of fish powder in school meals.

While the additional cost for substituting groundnut flour for fish powder seems quite small, it is worth considering at scale. Considering that 1,872,490 children receive meals from the school meals program in Malawi (including pre-, primary and secondary school) (46), if 2.5 g of fish powder per student were incorporated into the meal three times per week, this would result in an additional cost of USD 18,725 per week, or USD 675,000 for a 36-week school year. This represents about 4.8% of the total school meals budget (USD 13,964,063 in 2021) (46), highlighting the need to realize efficiencies in the fish powder value chain while still ensuring decent work for fisherfolk in order to ensure that procurement of fish powders for school meals is economically sustainable. The cost of fish powder production may be reduced by introducing improved technologies and improved processing procedures to maximize efficiency. However, this requires investment in technologies and infrastructure that may not be affordable for small-scale producers. Additional costs may also be incurred, such as increased storage and transportation capacity needs for the fish processors or due to involvement of middlemen in various stages of the supply chain such as distribution. Thus, the introduction of *usipa* powder in school meals would require investment throughout the fish value chain. Such investments could potentially have a ripple effect by improving quality and reducing loss in fish value chains that could benefit fish processors as well as domestic consumers more broadly and provide more markets (including export markets) for Malawian fish products.

Although the estimated cost of substituting fish powder for other ingredients in school meals would be a substantial national investment, it is expected that the benefits accrued from the addition of fish powder to school meals would have a substantial impact on children’s health and educational successes well into adulthood. By using data published in the Malawi Food Composition Table (57) for dried *usipa* powder and groundnut flour, we calculated the nutrient content of a meal containing 100 g maize flour, 25 g groundnut flour, and 30 g of leafy vegetables, based on a recipe from the HGSF recipe book (47). We then calculated the nutrient content of the same meal, with 2.5 g of fish powder substituted for 2.5 g of groundnut flour.

Based on the nutrient composition of recipes calculated using published food composition data, the porridge containing fish powder had greater content of protein (6%), thiamin (9%), riboflavin (22%), vitamin B6 (65%), niacin (34%),vitamin B12 (from 0 mcg to 0.95mcg), iron (44%), zinc (34%), calcium (8%), phosphorus (21%), and potassium (27%) per meal, and decreases in the content of vitamin E (−16%) and folate (−6%) in the meal. Although data was not available in the Malawi Food Composition table for fatty acids, iodine and vitamin A, it is expected that the content of these, particularly long-chained omega-3 polyunsaturated fatty acids Docosahexaenoic acid (DHA) and Eicosapentaenoic acid (EPA) would increase, as small whole fish are known to be a rich source (7). Fish powder produced from similar species in Zambia contained 73.5 mg of DHA in a 10 g serving (48) and 0.83–1.04 mcg of Retinol (vitamin A) per 100 g (49). While iodine content of freshwater fish species is known to be lower than marine fish species, freshwater species still contribute to iodine intake (50, 58). On the other hand, we must also note possible risks in the consumption of fish powder. Prior to the acceptability trials, chemical and microbiological analysis was conducted for all four *usipa* powders, which informed the research team on the selection of *usipa* powders to be tested by students in acceptability trials. Salt was used in the partial boiling step for parboiled usipa powder, however only 100 g of salt was used in parboiling 45 kg of fresh *usipa*, thus there is little estimated risk of increased sodium intake related to consumption of parboiled fish powder. Further research can provide a comprehensive review of food safety risk related to fish powder utilization.

The Nutrition Guidelines for School Meals stipulate that meals should include at least 350 kcal, 13 grams of protein, and micronutrients lacking in the diet, specifically vitamin A, iron, folate, and iodine, although the recommendations for micronutrients are not quantitative (51). The nutrition guidelines also note that the meal should include legumes, nuts or food from animals, as well as staples (e.g., maize or maize flour, etc.), fats, and vegetables or fruits. Currently, no animal-source food is included in school meals in Malawi (46). In addition to providing a greater amount of highly bioavailable micronutrients on their own, there are known enhancing effects on iron and zinc bioavailability from consuming animal-sourced foods such as fish alongside plant-sourced foods as part of a meal (52). Thus, our estimates of the nutrient benefits from enriching porridges with fish powder may be conservative.

Considering that over 60% of pre-school and school-aged children in Malawi are zinc-deficient, 21.7% of pre-school and 5.0% of school-aged children are iron-deficient, and more than 22% are anemic (53), the increased zinc and iron content of fish powder-enriched porridge may be a possible solution for improving zinc and iron intake. However, further studies are needed to determine if the increased intake of these minerals through fish powder-enriched school meals has a biologically significant effect on nutritional status of learners. While results from the Malawi Micronutrient Survey (2015/16) for vitamin B12 deficiency for pre-school and school-aged children are still pending, it is worth noting that 41% of women aged 15–49 years had depleted levels of vitamin B12 (53). The addition of animal-source foods such as fish powder to school meals is a potential source of vitamin B12 for children and adolescents, and can directly and indirectly contribute to greater zinc and iron absorption from the meal (52).

#### 4.4.2 Considerations for environmental sustainability

Here we consider issues relating to environmental sustainability (relating to the sustainability of the supply of raw materials, i.e., natural resources such as firewood and fish) in the case that the production of *usipa* powder is scaled up to serve all learners through school meal programs in Malawi. Based on conversion factors for the weight of fresh fish to the weight of dried fish powder recorded during this study (reported in section 3.3.1), we can calculate the quantity of fresh *usipa* required to serve 2.5 g of *usipa* powder to all learners benefiting from school meals in Malawi. Considering the same figure of 1,872,490 children receiving school meals in Malawi (including pre-, primary and secondary school) (46), if 2.5 g of fish powder per student were incorporated into the meal three times per week, 3,370,482 kg of *usipa* (based on fresh weight) would be required for a 36-week school year. Given that the annual supply of *usipa* in Malawi is 105,638 tonnes (based on live weight) (12), this represents 3.19% of total *usipa* production in the country. While this is a seemingly small percentage, it would be necessary to work with fisheries experts and the Malawi Department of Fisheries to ensure that the scaling up of fish powder production for school meals does not contribute to the depletion of usipa or other fish stocks in Malawian lakes.

Although the pan-roasted and parboiled *usipa* powders were the most acceptable for learners and had benefits in terms of food safety, scaling production of pan-roasted and parboiled *usipa* powder raises some concerns relating to environmental sustainability. The use of firewood for heat treatment in the processing of fish powder – whether it be pan-roasted, parboiled, or smoked fish powder – raises concerns over environmental sustainability. Improved, fuel-efficient technologies for pan-roasting, parboiling or smoking fish for fish powders are greatly needed if fish powder production is to be scaled up to meet the needs of school feeding programs in Malawi.

### 4.5 Limitations of this study

The estimates of the time cost for processing of fish powders and the cost of production of fish powder included in this article are only a snapshot, as this assessment was done with only one fish processing organization (Kacheta Cooperative, Monkey Bay, Mangochi, Malawi) at one point in time. This does not take into account the geographic nor temporal variability in the cost of fish and other raw materials, nor variance in costs related to labor or equipment rental. Further research is needed to gain a more representative understanding of the cost of fish powder production, taking into account the costs during different seasons and in different areas of Malawi where fish powder is produced.

While we present the data on time costs for processing fish powder and the cost of production per kilogram, this data should be interpreted carefully. For example, for some processes (smoking fish, parboiling fish), the amount of time needed would increase or decrease based on increased or decreased quantity of fish to process, while for other processes (such as sundrying), the amount of time will not vary as much based on the quantity of fish to process. However, more surface area to be used for sundrying may be needed to allow for greater quantities of fish to dry, and the amount of time needed for sundrying fish likely varies based on environmental conditions (i.e., warm, sunny weather versus cloudy weather). In addition, cost per kilogram and time for each processing cycle may be reduced with some improvements to processing procedures and capacity development. Furthermore, fisherfolk (and other food system actors) in Malawi are accustomed to using local measurement units in their trading activities, rather than scales with standardized measurement units (kg). Basins and buckets are used at landing sites and markets, and we found that there is variance in the weight of fish included (for example, a 5 L bucket of dried fish differed in weight depending on the fish product and how the fish was packed into the bucket). Thus, it is important to sensitize fisherfolk on the use of standardized measurements in order to make more accurate cost estimates to determine price of end products such as fish powder. The presentation of these estimates is only indicative, and further research is required to account for variability in the amount of time and costs associated with processing fish powder.

Lastly, analyses of the *usipa* powder destined for school meals to determine its full nutrient profile can provide insight into its fatty acid and vitamin composition. While the addition of nutrient content information from individual foods in the meal (as done here) gives an idea of the nutrient content of porridges served and tested in schools, it would be beneficial to conduct analysis of the nutrient content of cooked porridge with and without fish powder, to understand the effect of fish powder on the nutrient content of the whole meal. Despite these limitations, the study design allowed us to have a first-of-it’s kind analysis of the potential of fish powder in school meal programs in Malawi.

## 5 Conclusion

Adding animal-source food to school menus is one pathway to fight malnutrition and ensure food security. Fish powder has great potential to contribute to this agenda through school meal programs and should be promoted as an ingredient in school meals in Malawi. This study explored quality, convenience and affordability dimensions of the school food environment to explore the integration of fish powder into school meals from a holistic, multi-disciplinary approach for program design. The quality and convenience assessments resulted in high acceptability for schoolchildren of fish powders integrated in school meal recipes and convenience for school caterers and volunteers to prepare. However, there are efficiency gains that can be realized in fish value chains to improve upon convenience for fish powder producers, and issues relating to environmental and economic sustainability need to be considered when scaling up the use of fish powders in school meal programs.

To further support the integration of safe, nutritious, and affordable fish products into school menus in a sustainable way, we recommend consideration of the acceptability of these products when being integrated into local recipes. Even though all recipes were liked by learners, results shows that some were preferred to others, and that higher acceptability of the porridge was associated with lower plate waste. The impact of introducing new food to the school meals should mitigate the risk of reducing overall food and nutrient intake of the learners benefiting from school meals. Thus, the selection of the right products will maximize the impact of school meals for nutrition, while contributing to reduced food loss and waste, which are both of utmost importance to meeting the Sustainable Development Goals by 2030. At the national level, we recommend that policymakers allocate the necessary budget to finance school meal programs, build capacity of cooperatives and organizations of food producers to supply local foods to school meal programs, and invest in small-scale infrastructure and technologies that empower these producers to scale up their activities, increase efficiency and reduce production costs. Given recent shifts in the funding environment for international development and aid, improving domestic food supply has potential to contribute to a more sustainable and nutritious school meal program.

This study is the first of its kind to provide an in-depth multidimensional analysis of the: (1) availability, affordability, quality, and convenience of incorporating fish powder into school meal programs; (2) costs associated with producing locally sourced fish powders; and (3) the trade-offs across these dimensions and associated issues of sustainability across the fish powder value chain in Malawi. By grounding this study in the food environment theoretical framework, it provides insights that go beyond supply and demand of fish products to better target interventions for improving nutrition. Given the limitations enumerated in our study, we recommend future research and capacity building of local fisherfolk to record costs throughout the fish powder production cycle to gain a better understanding of costs across seasons and geographic locations. We also recommend conducting nutrient analysis to provide full nutrient profiles of different types of *usipa* fish powders and different types of *usipa* powder-enriched porridges, and research into sustainable sources of energy for parboiling and pan-roasting of fish powders.

## Data Availability

The raw data supporting the conclusions of this article will be made available by the authors, without undue reservation.
