# Supplementary material for: Exploring the integration of fish powder in school meal programs in Malawi through a food environment lens: acceptability, affordability, and convenience
Source: Front Nutr. 2025 Jun 23;12:1605540. doi: 10.3389/fnut.2025.1605540 (PMC12229799; doi:10.3389/fnut.2025.1605540)
Supplement: Supplementary file 1 [file Table_1.docx]

## Supplementary Materials

*S1. School Recipes and Modified Recipes for Acceptability Trials in Mangochi and Dowa – recipes served 120 students each*

| **School Recipes - Mangochi** | | **Modified Recipes for Acceptability Trials - Mangochi** | | **School recipes - Dowa** | | **Modified Recipes for acceptability trials - Dowa** | |
| --- | --- | --- | --- | --- | --- | --- | --- |
| **Porridge Name** | **Ingredients** | **Porridge Name** | **Ingredients** | **Porridge** | **Ingredients** | **Porridge** | **Ingredients** |
| MAIZE-GROUNDNUT FLOUR PORRIDGE | - 11kg maize flour (approx. 92g / learner) - 1kg groundnut flour (approx. 8g / learner) - 70L water - 250g salt - 500g sugar | PARBOILED *USIPA* POWDER-ENRICHED MAIZE-GROUNDNUT FLOUR PORRIDGE | - 11kg maize flour (approx. 92g / learner) - 1kg groundnut flour (approx. 8g / learner) - 300g parboiled *usipa* powder (approx. 2.5g / learner) - 70 L water - 250g salt - 500g sugar | MAIZE-GROUNDNUT FLOUR PORRIDGE | - 10kg maize flour (approx. 83g / learner) - 2.4kg groundnut flour (approx. 20g / learner) - 70L water - 250g salt - 500g sugar | PARBOILED *USIPA* POWDER-ENRICHED MAIZE-GROUNDNUT FLOUR PORRIDGE | - 15.7kg maize flour (approx. 130g / learner) - 1.4kg groundnut flour (approx. 12g / learner) - 428g Parboiled *usipa* powder (approx. 3.5g / learner) - 100 L water - 357g salt - 714g sugar |
|  |  | PAN-ROASTED *USIPA* POWDER-ENRICHED MAIZE-GROUNDNUT FLOUR PORRIDGE | - 11kg maize flour (approx. 92g / learner) - 1kg groundnut flour (approx. 8g / learner) - 300g pan-roasted *usipa* powder (approx. 2.5g / learner) - 70 L water - 250g salt - 500g sugar |  |  | PAN-ROASTED *USIPA* POWDER-ENRICHED MAIZE-GROUNDNUT FLOUR PORRIDGE | - 15.7kg maize flour (approx. 130g / learner) - 1.4kg groundnut flour (approx. 12g / learner) - 428g pan-roasted usipa powder (approx. 3.5g / learner) - 100 L water - 357g salt - 714g sugar |
|  |  |  |  |  |  | SUNDRIED *USIPA* POWDER-ENRICHED MAIZE-GROUNDNUT FLOUR PORRIDGE | - 14kg maize flour (approx. 117g / learner) - 1.3kg groundnut flour (approx. 11g / learner) - 386g sundried *usipa* powder (approx. 3.22g / learner) - 90 L water - 321g salt - 643g sugar |
|  |  |  |  |  |  |  |  |
|  |  |  |  |  |  |  |  |
|  |  |  |  |  |  |  |  |
|  |  |  |  |  |  |  |  |
|  |  |  |  |  |  |  |  |
| **MAIZE-SOYA FLOUR PORRIDGE (CONTROL)** | - 9kg maize flour - 3kg soya flour - 70 L water - 250g salt - 500g sugar | PARBOILED *USIPA* POWDER-ENRICHED MAIZE-SOYA FLOUR PORRIDGE | - 9kg maize flour - 3kg soya flour (approx. 25g / learner) - 300g parboiled *usipa* powder (approx. 2.5g / learner) - 70 L water - 250g salt - 500g sugar | **MAIZE-SOYA FLOUR PORRIDGE (CONTROL)** | - 12.9kg maize flour (approx. 108g / learner) - 2.7kg soya flour (approx. 23g / learner) - 90L water - 321g salt - 643g sugar | PARBOILED *USIPA* POWDER-ENRICHED MAIZE-SOYA FLOUR PORRIDGE | - 14kg maize flour (approx. 117g / learner) - 3kg soya flour (approx. 25g / learner) - 428g parboiled *usipa* powder (approx. 3.5g / learner) - 100 L water - 357g salt - 714g sugar |
|  |  | PAN-ROASTED *USIPA* POWDER-ENRICHED MAIZE-SOYA FLOUR PORRIDGE | - 9kg maize flour - 3kg soya flour - 300g pan-roasted *usipa* powder (approx. 2.5g / learner) - 70 L water - 250g salt - 500g sugar |  |  | PAN-ROASTED *USIPA* POWDER-ENRICHED MAIZE-SOYA FLOUR PORRIDGE | - 12.9kg maize flour (approx. 108g / learner) - 2.7kg soya flour (approx. 23g / learner) - 386g pan-roasted *usipa* powder (approx. 3.22g / student) - 90 L water - 321g salt - 643g sugar |
|  |  |  |  | School porridge: Corn (maize)-soya blend (CSB) porridge | Pre-mix of maize and soya beans mixed with sugar, vitamins, and minerals (quantities obscure) and about 300-350g of salt is added | SUNDRIED *USIPA* POWDER-ENRICHED MAIZE-SOYA FLOUR PORRIDGE | - 12.9kg maize flour (approx. 108g / learner) - 2.7kg soya flour (approx. 23g / learner) - 386g sundried *usipa* powder (approx. 3.22g / learner) - 90 L water - 321g salt - 643g sugar |
|  |  |  |  |  |  |  |  |
|  |  |  |  |  |  |  |  |
|  |  |  |  |  |  |  |  |
|  |  |  |  |  |  |  |  |
|  |  |  |  |  |  |  |  |

S2. Data collected during scoping study – enrolment rates and amount spent per week on school meals

| **District** | **School** | **Enrolment (n. students)** | **Funding Source** | **Amount Spent / week (MK)** | **Amount spent / student / meal (5 meals / week) (MK)** | **Amount spent / student / meal (5 meals / week) (USD)** |
| --- | --- | --- | --- | --- | --- | --- |
| Dowa | Chilima | 875 | Mary’s Meals | MK 480,000.00 | MK 109.71 | USD 0.06 |
|  | Dowa 1 | 2430 | Mary’s Meals | MK 1,200,000.00 | MK 98.77 | USD 0.06 |
|  | Kafumphe LEA | 982 | Mary’s Meals | MK 550,000.00 | MK 112.02 | USD 0.06 |
|  | Namatunje | 768 | Mary’s Meals | MK 500,000.00 | MK 130.21 | USD 0.07 |
| Mangochi | Mbwadzulu | 2107 | WFP | MK 2,000,000.00 | MK 189.84 | USD 0.11 |
|  | Chilumba | 956 | Community-based | MK 125,000.00 | MK 26.15 | USD 0.01 |
|  | Mangombo | 1533 | WFP | MK 1,954,316.00 | MK 254.97 | USD 0.15 |
|  | Monkey Bay | 1652 | WFP | MK 615,108.00 | MK 74.47 | USD 0.04 |
|  | Mtakataka | 1685 | WFP | MK 2,066,600.00 | MK 245.29 | USD 0.14 |
|  | Namazizi | 761 | WFP | MK 86,626.00 | MK 22.77 | USD 0.01 |
|  | St Louis | 1149 | WFP | MK 839,931.00 | MK 146.20 | USD 0.08 |
| Salima | Chambawala | 268 | Mary’s Meals | MK 200,000.00 | MK 149.25 | USD 0.09 |
|  | Kacherenje | 1105 | WFP | MK 1,222,004.00 | MK 221.18 | USD 0.13 |
|  | Kasache | 840 | Mary’s Meals | MK 450,000.00 | MK 107.14 | USD 0.06 |
|  | Mikute | 2391 | WFP | MK 294,683.00 | MK 24.65 | USD 0.01 |
|  | Parachute | 1306 | WFP | MK 100,000.00 | MK 15.31 | USD 0.01 |
|  | Senga Bay | 2127 | WFP | MK 1,868,850.00 | MK 175.73 | USD 0.10 |
| Karonga | Wovwe Primary School | 563 | Community-based | no data | no data | no data |
|  | Ulaha Primary School | 468 | Community-based | no data | no data | no data |
|  | Thukutu Primary School | 214 | Community-based | no data | no data | no data |
|  | Thunduti Primary School | 425 | Community-based | no data | no data | no data |
|  | Ndindo Primary School | 315 | Community-based | MK 150,000.00 | MK 95.24 | USD 0.05 |
|  | Mayoka Primary School | 834 | Community-based | MK 270,000.00 | MK 64.75 | USD 0.04 |
